# Calculating the mean time to capture for tethered ligands and its effect on the chemical equilibrium of bound ligand pairs

**DOI:** 10.1016/j.dib.2016.05.050

**Published:** 2016-05-28

**Authors:** Lu Shen, Caitlin G. Decker, Heather D. Maynard, Alex J. Levine

**Affiliations:** a Department of Chemistry & Biochemistry, University of California, Los Angeles, CA 90095, USA; b Department of Physics & Astronomy, University of California, Los Angeles, CA 90095, USA; c Department of Biomathematics, University of California, Los Angeles, CA 90095, USA; d California Nanosystems Institute, University of California, Los Angeles, CA 90095, USA

**Keywords:** Ligand binding

## Abstract

We present here the calculation of the mean time to capture of a tethered ligand to the receptor. This calculation is then used to determine the shift in the partitioning between (1) free, (2) singly bound, and (3) doubly bound ligands in chemical equilibrium as a function of the length of the tether. These calculations are used in the research article Fibroblast Growth Factor 2 Dimer with Superagonist in vitro Activity Improves Granulation Tissue Formation During Wound Healing (Decker et al., in press [1]) to explain quantitatively how changes in polymeric linker length in the ligand dimers modifies the efficacy of these molecules relative to that of free ligands.

## Specification table

**Table:** 

Subject area	*Physics, Chemistry*
More specific subject area	*Statistical mechanics and polymer physics*
Type of data	*Calculations*
How data was acquired	*A cell viability assay was conducted with human dermal fibroblast cells*
Data format	*Text and figure*
Experimental factors	*Effect of linker/tether length between two FGF2 proteins on cellular activity*
Experimental features	*Linker lengths were varied between 118 and 20,000 Da (N=1–454)*
Data source location	*Los Angeles, CA*
Data accessibility	*Data is within this article*

## Value of the data

• There are a number of ligands that bind to cell receptors as dimers or multimers. These include transcription factors and enzymes. For example the G-protein coupled receptor family, which includes receptors for dopamine, somatostatin, and bradkykinin all exhibit dimer-dependent activity. This for reason, future drug development may involve more broadly the use of tethered ligands to enhance the efficacy of ligand binding, as was explored in the research article [1]. Thus, the fundamental analysis of the chemical kinetics of tethered ligand binding, which we present here, is of general interest for drug design.
• In this data in brief we present an analysis of the chemical kinetics of tethered ligand binding in a manner generally applicable to all studies of tethered ligand binding since it relies only on fundamental statistical physics.
• The principal effects of the tether are to first keep a second ligand nearby the first and second to provide a harmonic potential pulling that second ligand towards the receptor. These two effects shorten the mean time to capture for the second ligand, particularly at low ligand concentrations.
• We calculate the effect of the length of polymeric tether on the equilibrium concentration of bound dimers. This result could be generally applicable to all tethered-dimer binding problems.
• We present the details of the calculation with sufficient detail to allow other workers to modify it as necessary for general problems related to tethered molecule binding problems.
• We show data on the effect of cellular metabolic activity versus linker length along with a fit to our calculations.

## 1 Data

We calculate the mean time to capture of a ligand tethered by a Gaussian polymer coil in part one below. We then use this calculation in part two to determine how the length of the polymer controls the chemical equilibrium between free and bound ligands. Finally, we present data on cellular metabolic activity versus linker length and compare it to the theory presented. We conclude with a discussion of the fitting parameters used.

## 2 Experimental design, materials and methods

### 2.1 Mean time to capture for a tethered ligand

We present the calculation of the mean time to capture for a tethered and an untethered ligand. We treat the tether as a Gaussian coil in the weakly stretched limit resulting in a Hookean effective potential between the ligand and its binding site. We treat the binding site as an isolated spherical volume of radius a, which has dimensions characteristic of the receptor, a~10nm. One may consider an ensemble of such ligands distributed about the binding site with concentration field $c(\vec{x},t)$. The membrane to which the receptor is bound is impenetrable to the ligands and thus introduces a Dicherlet boundary condition requiring the normal component of the concentration current to vanish at the membrane. This extra boundary condition complicates the solution, but should not change the fundamental scaling behavior of the answer, so we neglect it here. Accordingly, we treat the binding site as an isolated volume centered at the origin of coordinates in a system with spherical symmetry. This ligand undergoes a biased random walk towards the binding volume as it is pulled by the polymeric tether. To calculate the mean time to capture we imagine a related steady-state advection diffusion problem. Particles are released at a constant rate on the surface of a sphere of radius *R* centered on the capture sphere of radius a<R. This fixes the particle concentration there to a fixed (and ultimately irrelevant) value. These particles are annihilated when they contact the capture sphere. There is also an outer boundary sphere with reflecting boundary conditions; particles reaching this outer boundary reflect off of it and are thus confined in the space between the inner capture sphere and the outer boundary sphere: $a<\;r<R^{*}$. The role of this outer boundary is to mimic the effect of the maximum extension of the polymeric tether: R*=*Nb*, where *N* is the polymerization index and bis the Kuhn length [2]. We ignore the nonlinear elasticity of the polymer in the strongly stretched regime $R\sim R^{*}$. As a result, our answer should overestimate the mean time to capture. But since strongly stretched states of the polymer are exponentially rare $\kappa R^{*2}\gg k_{B}T$, we expect the magnitude of the error is small, and from now on, we will work in the units that the Boltzmann׳s constant is unity $k_{B}=1$.

By solving this spherically symmetric advection diffusion problem in three dimensions, we determine (1) the steady-state number of particles in the system and (2) the rate at which they are being annihilated on the surface of the capture sphere. The ratio of the number of particles in the system to the annihilation rate is then the mean lifetime of the particles in the system [3], [4]. This is equal to the mean time for a tethered particle, initially at a distance R from the origin, to first reach the capture sphere. We take this initial distance to be the root mean square end-to-end distance of the polymer in thermal equilibrium to then estimate the mean time to capture of the free ligand attached to the polymeric tether. This approximation neglects both the distribution of such end-to-end distances in equilibrium and the potential effect of the cell membrane surface in perturbing the equilibrium distribution of those end-to-end distances. In principal, both effects could be studied in a more sophisticated treatment of the first passage time calculation, but we suspect that these effects are small compared to two main ones incorporated in our more simple treatment. Namely, (1) the effect of the tether brings a second ligand near to the receptor upon the binding of the first of the tethered ligands, and (2) the polymeric tether provides a Hookean spring, enhancing the likelihood of finding the ligand close to the already bound one.

#### 2.1.1 The advection diffusion problem

As outlined above, we consider following the steady-state diffusion problem with spherical symmetry. Particles are release at a fixed radius R. While attached to Hookean spring anchored the origin of coordinates, they move between an outer boundary of radius $R^{*}>R$ with reflecting boundary conditions and an inner boundary of radius a<R representing the capture sphere of the already bound ligand/receptor pair. The reflecting outer boundary is taken to be the maximum extension of the polymeric tether. The role of the tether (other than providing this outer boundary condition) is to give an effective parabolic potential in which the tethered ligand diffuses.

Given such boundary conditions discussed above, the ligand concentration current $\vec{J}$ is given in terms of the concentration c where we have introduced the ligand diffusion constant D=Tμ with mobility μ and the (polymeric) spring constant κ.

$$\vec{J}=-D\nabla c-\kappa \mu \vec{r}c$$

These particles are annihilated upon contact with the binding sphere (the spherical zone of radius *a* centered on the origin of coordinates) so that

c(a)=0

To balance in steady state this loss of particles, which is equal to the total flux of particles *into* the capture sphere

$$F=4\pi a^{2}\nabla J\cdot {\hat{r}|}_{r=a}$$

We inject new particles at the radius *R* so that the concentration there is fixed in time:

c(R)=1

We have set this steady-state concentration to unity without loss of generality. Finally, at the outer boundary, we require a zero flux boundary condition

$$\vec{J}\cdot {\hat{r}|}_{r=R^{*}}=0$$

so that no particles may escape to larger radii. Thus, in this steady-state system the total number of particles is

$$N=\int_{a\leq \left|\vec{x}\right|\leq R^{*}}d^{3}\vec{x}c(\vec{x})$$

By taking the ratio of the number of particles (tethered ligands) in this steady-state system to their annihilation rate, both given above, we determine their mean life-time in the system

$$T(R,\kappa )=\frac{N}{F}$$

As a final step we set the initial radius of the particles to be the rms end-to-end distance of the polymer $R_{rms}=\sqrt{N}b$ to obtain the mean time to capture for the second ligand. Since the maximum distance R*=*Nb* and the polymeric spring constant

$$\kappa (N)=\frac{3T}{b^{2}N}$$

are also determined by the polymerization index *N*, we may write the final mean time (using the above spring constant) to capture solely in terms of *N*, a characteristic diffusion time, and the ratio of the capture radius to the Kuhn length

$$\tau \left(N,\frac{a}{b}\right)=\tau_{0}\overset{¯}{T}(R_{rms},\kappa (N))$$

where we have introduced a nondimensionalized capture time $\bar{T}$ and a fundamental time scale

$$\tau_{0}=a^{2}/D$$

representing the typical time scale for a ligand to diffuse a distance equal to the radius of the capture sphere. Since we expect both the Kuhn length and capture radius of a cell membrane bound receptor to be a molecular size, we expect a~b. To simplify the results, we take them to be equal in the following.

#### 2.1.2 Stead-state concentration profiles

The solution to the steady-state concentration profile results requiring the divergence of the concentration current to vanish, which requires: $\nabla \cdot \vec{J}=0$. Given the imposed spherical symmetry of the problem, this reduces to one-dimensional differential equation for the radial dependence of the steady-state concentration profile:

$$\partial_{r}^{2}c(r)+\left(\frac{2}{r}+\frac{\kappa }{T}r\right)\partial_{r}c(r)+\frac{3\kappa }{T}c(r)=0$$

It is helpful to introduce a dimensionless independent variable defined by

$$x=r\sqrt{\frac{\kappa }{T}}=r/r_{0}$$

In terms of this variable the differential equation for the concentration field takes the form

$$c\prime \prime (x)+\left(\frac{2}{x}+x\right)c\prime (x)+3c(x)=0$$

The physical meaning of this variable transformation is that we now work in distance units so that the work done to stretch the polymeric spring to x=1, or $\kappa r^{2}$, is equal to thermal energy T. The solution to the above differential equation is given by the linear combination

$$c(x)=Ae^{-\frac{x^{2}}{2}}+Bf(x)$$

where the function *f*(*x*)

$$f(x)=\sqrt{\frac{\pi }{2}}e^{-\frac{x^{2}}{2}}erfi\left(\frac{x}{\sqrt{2}}\right)-\frac{1}{x}$$

may be written in terms of the imaginary error function [5].

We now determine an inner and outer solution where the inner solution satisfies the boundary conditions at r=a,R and the outer solution satisfied the boundary conditions at $r=R,R^{*}$. Of course, this implies a discontinuity in the radial derivative of the concentration field at r=R, which is physically understandable as a delta-shell source of particles injected into the system to maintain the steady-state concentration field.

For the inner solution we find the coefficients are

$$A_{inner}=\frac{-f(\bar{a})e^{\frac{{\bar{a}}^{2}}{2}}}{f(\bar{R})-f(\bar{a})\exp[-\left({\bar{R}}^{2}-{\bar{a}}^{2}\right)/2]}B_{inner}=\frac{1}{f(\bar{R})-f(\bar{a})\exp[-\left({\bar{R}}^{2}-{\bar{a}}^{2}\right)/2]}$$

In the above equations and hereafter it is convenient to introduce dimensionless radii (denoted by an overbar) by scaling these distances by $r_{0}$ defined above, $\bar{a}=a/r_{0}$ and $\bar{R}=R/r_{0}$, with $r_{0}=b\sqrt{\frac{N}{3}}$.

For the outer solution we find that

$$A_{outer}=e^{{\bar{R}}^{2}/2}B_{outer}=0$$

This simple result may be understood as follows. Of the two linearly independent solutions to the differential equation, the first one has zero current everywhere as may be checked by the fact that it is equal to the equilibrium probability distribution for a particle in a parabolic potential. As an equilibrium solution, it must give zero current. Thus the zero current boundary condition at the outer boundary forces $B_{outer}=0$. The value of the coefficient of the first solution is then immediately clear from the concentration condition at the shell of particle injection.

Taking the above results we compute the rate of particle annihilation at the surface of the capture sphere to be

$$F=4\pi DB_{inner}r_{0}$$

We compute the total number of particles in the steady-state system from the integral of the steady-state concentration field over the allowed range between r=a and $r=R^{*}$. The result is somewhat complex

$$N=4\pi r_{0}^{3}\left[A_{inner}\Delta_{1}+B_{inner}\Delta_{2}+A_{outer}\Delta_{3}-\frac{B_{inner}}{2}({\bar{R}}^{2}-{\bar{a}}^{2})\right]$$

but can be written compactly in terms of three dimensionless integrals:

$$\Delta_{1}=\int_{\bar{a}}^{\bar{R}}z^{2}e^{-z^{2}/2}dz\;\Delta_{2}=\int_{\bar{a}}^{\bar{R}}z^{2}\left[f(z)+\frac{1}{z}\right]dz\;\Delta_{3}=\int_{\bar{R}}^{{\bar{R}}^{*}}z^{2}e^{-z^{2}/2}dz$$

Forming ratio of N/F to get the mean time to capture for the ligand we write

$$T(\bar{R})=\frac{r_{0}^{2}}{DB_{inner}}\left[A_{inner}\Delta_{1}+B_{inner}\Delta_{2}+A_{outer}\Delta_{3}-\frac{B_{inner}}{2}({\bar{R}}^{2}-{\bar{a}}^{2})\right]$$

**Fig. 1:**
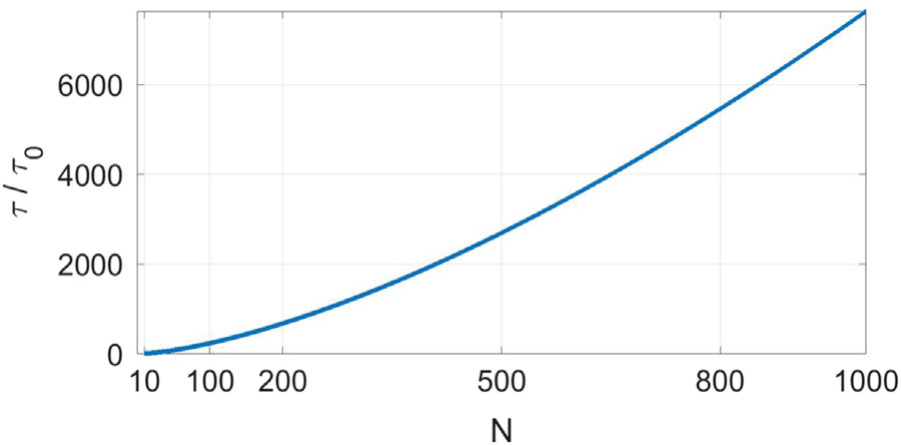
The mean time to capture in units of the diffusion time $\tau_{0}=a^{2}/D$ as a function of the polymerization index N. The large and small *N* limits of this function are discussed in the text.

The final result for the mean time for second ligand capture after the binding of the first tethered ligand is given by the above result evaluated at ${\overset{¯}{R}}_{rms}=\sqrt{N}b/r_{0}$. We also use the polymeric spring constant κ(N) defined above, and set the maximum length of the tether to be ${\overset{¯}{R}}^{*}=Nb/r_{0}$. This result scaled by $\tau_{0}$ is plotted in Fig. 1 as a function of the polymerization index *N*.

$$\tau (N)=\tau_{0}\left[\frac{T(\sqrt{N}b/r_{0})}{\tau_{0}}\right]$$

The small *N* limit of the mean time to capture, τ→0 as *N* approaches one from above, is trivially true in that the starting length of the tether approaches the Kuhn length b, which has been set to the capture radius. The Gaussian coil model of the polymer is clearly inappropriate in this case and our neglect of the size and shape of the ligands is similarly unjustifiable. Excluded these details that must become important in the limit of extremely short tethers, we do expect the mean time to capture to be a monotonically increasing function of the polymerization index *N*.

In the limit of high molecular weight tethers, where our analysis is applicable, one may check that the mean time to capture approaches a power-law in *N*:

$$\tau (N)\sim \tau_{0}N^{3/2}$$

The increase in mean time to capture results physically from two separate effects. First, the initial distance between the ligands grows as *N*^1/2^. Second, the polymeric spring constant decreases as *N*^−1^. The result above implies that the mean time to capture diverges as the polymerization index goes to infinity. This is unphysical at any finite concentration of ligands. For extremely long tethers and a finite concentration of ligands, we expect that the time for the second ligand binding will be cut off at large *N* when the mean time to capture for the second ligand in the tethered pair exceeds the mean time for capture for a free ligand (or one ligand in a different tethered pair). If the number density of ligands is $c_{\infty }$, implying a mean spacing between ligands of $c_{\infty }^{-1/3}$ then the competitive binding of the untethered ligands will dominate at tether lengths larger than $\sqrt{N^{*}}b\sim {c_{\infty }}^{-1/3}$ leading to an upper bound on the mean time to capture

$$\tau_{\max}=\tau (b^{-3}/c_{\infty })\sim \tau_{0}{\left(b^{3}c_{\infty }\right)}^{-1/2}$$

We expect that at relevant concentrations of the ligand, $\tau_{max}>>\tau \left(N\right)$. Otherwise, the effect of the tether on the binding kinetics and the steady-state concentration of doubly bound ligands will be negligible.

### 2.2 Polymer tether effects on the binding kinetics

**Fig. 2:**
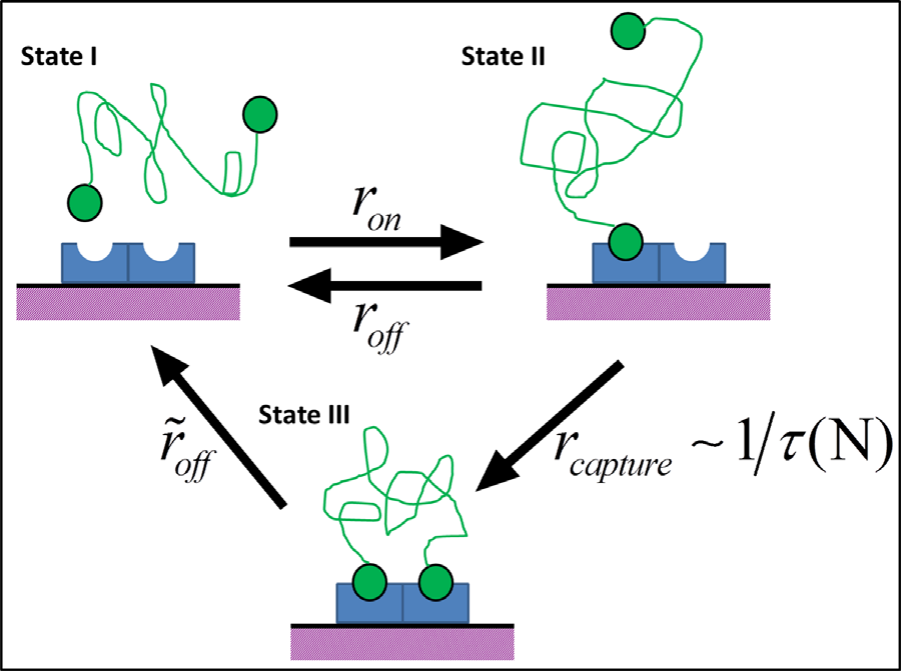
A schematic representation of the tethered ligand binding process. In state I the free ligand pair is shown tethered by the (green) random coil polymer that produces an effective Hookean potential. This tethered ligand pair binds to a membrane bound receptor (blue) on the cell surface (purple) with on and off rates $r_{on}$, $r_{off}$ respectively. The transition from the singly bound state (II) to the signaling, doubly-bound one (III) is assumed to occur with a rate equal to the inverse of the mean capture time determined above: $r_{capture}$. The doubly bond state is stabilized relative to the singly bound one, but has a small off rate ${\overset{˜}{r}}_{off}<<r_{on},r_{off}$. The backwards rate from state III to state II is assumed to even slower and it thus neglected, although this is not an essential simplification of the model.

We now consider a simple model for the binding kinetics of the tethered ligand to the receptor. The binding of the single ligand is weak, but with the formation of the additional ligand/receptor bond, the complex becomes strongly bound. We propose a simple three state kinetic model of the process. The states are (I) free tethered ligands, (II) singly bound tethered ligands, and (III) doubly bound tethered ligands. A schematic representation of the three states and the allowed transitions is shown in Fig. 2.

As calculated above, the advantage of the tether is to decrease the time (i.e., increase the reaction rate) of the transition between states II and III, and thereby suppress the competing reaction of II→I. To examine how this affects the concentration of doubly bound linkers, we write a set of three coupled rate equations for time evolution of the probabilities of observing any of the three states of linker binding, $P_{\alpha }(t)$, α=I,II,III. In terms of the interconversion rates (defined below) these equations take the form of

$$\overset{•}{P_{I}}=-P_{I}(t)r_{on}+P_{II}(t)r_{off}+P_{III}(t){\overset{˜}{r}}_{off}\overset{•}{P_{II}}=P_{I}(t)r_{on}-P_{II}(t)[r_{off}+r_{capture}]\overset{•}{P_{III}}=P_{II}(t)r_{capture}-P_{III}(t){\overset{˜}{r}}_{off}$$

The first equation determines the time rate of change for the probability of observing free (unbound) tethered linkers in terms of the rate at which singly bound linkers bind $r_{on}$, unbind $r_{off}$ and the (much slower) rate at which doubly bound linkers unbind ${\overset{˜}{r}}_{off}<<r_{off}$. The second two equations can be interpreted similarly; Fig. 2 provides a simple pictorial representation of the full set of these equations.

The contribution of the tether to the binding kinetics is found entirely in the transition from state II to state III; $r_{capture}=1/\tau \left(N\right)$ provides a faster rate of second linkers binding than would otherwise occur.

Solving for the steady-state probabilities (by setting the time derivatives on the right hand side of the above system of equations to zero), one immediately finds that ratio of the probability of observing a doubly bound linker to the receptor pair to observing free linkers is

$$\frac{P_{III}}{P_{I}}=\frac{r_{on}}{{\overset{˜}{r}}_{off}}\left[\frac{1}{1+\tau (N)r_{off}}\right]$$

This is equal to the ratio of the concentration of doubly bound linkers to free linkers in the steady state.

To quantify the enhancement of binding due to the presence of the tether, it is useful to compare the concentration of doubly bound tethered linkers with the doubly bound untethered linkers, both in thermal equilibrium. To examine the case of untethered linkers, we simply replace $r_{capture}=1/\tau \left(N\right)$ with $r_{on}$. Thus, the enhancement of doubly bound linkers ℜ is

$$ℜ=\frac{1+K_{d}}{1+r_{on}\tau (N)K_{d}}$$

when written in terms of the dissociation constant of the single ligand receptor bond (divided by the concentration of membrane bound receptors) and on rate of single ligands $r_{on}$.

Stating this in another way, a concentration $c_{0}$ of tethered linkers in solution will doubly bind to the membrane-bound receptors (and presumably generate the same effective cell signaling) as a higher concentration $ℜc_{0}$ of *untethered* linkers. Note that ℜ>1 and represents an enhancement factor of the effective concentration so long as $r_{on}\tau (N)<1$. The mean time to capture for the tethered linker must be shorter than the mean time for capture of a free one. As noted above, the growth of τ(N) with polymerization index (and thus the decrease of the enhancement factor ℜ) is cut off by the competitive binding of free ligands. We see that the *concentration enhancement factor* can range between:

$$1\leq ℜ\leq 1+K_{d}$$

Larger enhancement factors are only possible if one assumes that the polymer tether also changes either the rate of attachment in some other way, i.e., in orienting the ligands appropriately for binding (an effect we do not consider in this model) or by directly changing the binding energy of the complex. We consider the latter possibility to be highly remote.

### 2.3 Polymer tether length versus cellular metabolic activity

**Fig. 3:**
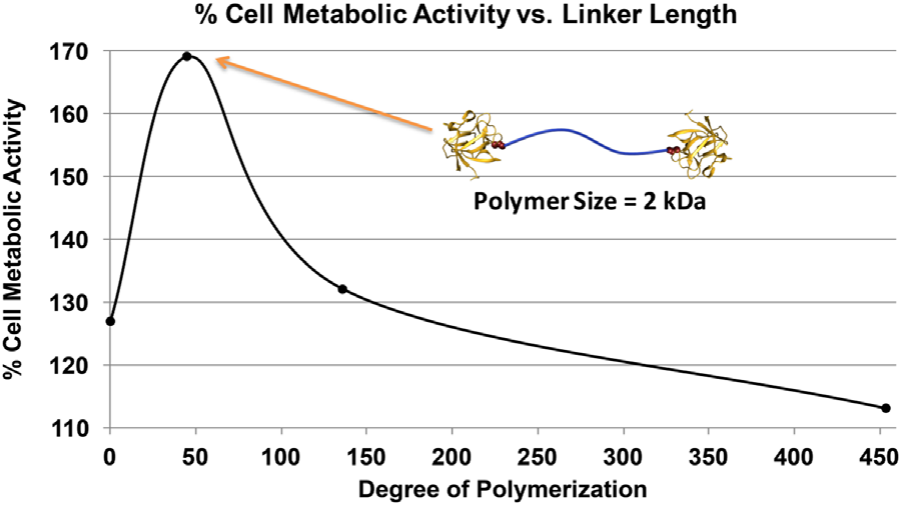
Percent metabolic activity of human dermal fibroblasts normalized to blank medium versus degree of polymerization (*N*, number of monomer repeat units) in the covalent tether between two FGF2 molecules. The highest percent cell metabolic activity was observed for FGF2 dimer with a linker length of 2 kDa.

The principal point of our analysis is the result. But this concentration enhancement factor is only indirectly measured in our experiments, which determine the cellular metabolic activity enhancement in the presence of tethered dimers (at fixed concentration) as a function of tether length. These data are shown in Fig. 3. Cellular metabolic activity is measured in human dermal fibroblast cells via the CellTiter-Blue^®^ assay after adding FGF2 dimer with different PEG tether lengths. Each result is normalized to blank medium only as the control group.

In order to compare these data with the theoretical calculation, we need to make a number of assumptions, as outlined here. First, we associate the enhancement of cellular metabolic activity with the enhancement of the bound dimers on the cell membrane by assuming a linear relationship between the concentration of bound dimers and the increase of metabolic activity *M* over its basal value (100%). Given that cell signaling is quite possibly nonlinear, this assumption may be invalid. We adopt it, however, in order to minimize the number of fitting parameters in our model. Thus we express the metabolic activity enhancement (as a percentage)

$$\Delta M=\frac{M-M_{0}}{M_{0}}=\alpha (ℜ-1)$$

as being proportional to the enhancement of the efficacy of tethered ligand binding with an unknown proportionality constant *α*. Treating the on rate also as a free parameter we attempt to fit the data with two parameters:

$$\Delta M_{theory}=\frac{\beta -\gamma N^{3/2}}{1+\gamma \;N^{3/2}}.$$

**Fig. 4:**
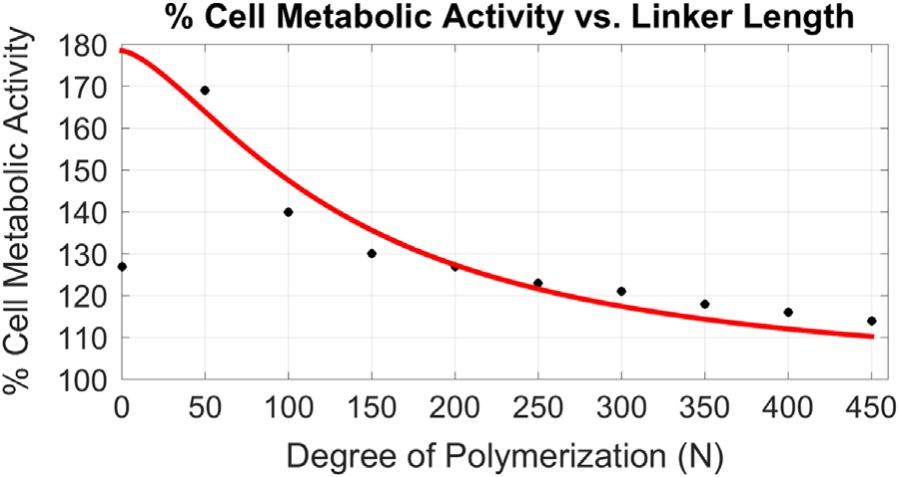
The (red) theoretical curve $\Delta M_{\mathrm{theory}}(N)$ (with two fitting parameters adjusted to achieve the best fit when excluding the point at *N*=1) compared to the experimental results – see Fig. 3 – represented by nine black circles taken from the interpolation function shown in Fig. 3. The best fit is achieved with β=78.5 and γ=0.00063.

Here we adjust the constants β, γ to fit the data. The results are shown by the red line in Fig. 4, the fitting was based on several data points and using the least squares method. Comparing this fit to the data we observe that one may understand the increase in metabolic activity by the increase in dimerized ligand binding associated with shortening the polymeric tether. We find that the curvature of the fit agrees reasonably well with the data, suggesting that the proposed mechanism for the tether׳s enhancement of dimerized-ligand binding is supported by these data. We also observe that one cannot account for the rapid decrease in metabolic activity with a further reduction of molecular weight of the polymeric tether below 2 kDa. As discussed earlier, for these shorter tethers the model assumptions of Gaussian coil tethers and no steric interactions between the tether and the ligands (or between the two ligands) must become invalid. The fact that these effects are not evident until the point of maximum metabolic activity suggests that the omitted steric interactions become dominant at this point. In fact, it is reasonable to suppose that the point at which steric interactions begin to dominate is precisely the point at which the binding enhancement of associated with short tethers begins to diminish. Only future molecular dynamics simulations can directly test this point.

Finally, we note that a more direct test of the theory outlined here requires a direct measurement of the concentration of bound dimers as a function of tether length. Such experiments, presumably in an in vitro membrane system may be possible in the future.
